# 18F-FDG PET-CT- vs. CT-Based Radiotherapy Treatment Planning for Head and Neck Cancer

**DOI:** 10.3390/life16020263

**Published:** 2026-02-03

**Authors:** Admir Mulita, Eleni Bekou, Pipitsa Valsamaki, Ioannis M. Koukourakis, Francesk Mulita, Elias Liolis, Athanasios Zissimopoulos, Alexandra Giatromanolaki, Michael I. Koukourakis

**Affiliations:** 1 Department of Radiotherapy/Oncology, School of Medicine, Democritus University of Thrace, 68100 Alexandroupolis, Greece; admirm@hotmail.gr; 2 Medical Physics Laboratory, School of Medicine, Democritus University of Thrace, 68100 Alexandroupolis, Greece; ebekou@med.duth.gr; 3 Department of Nuclear Medicine, School of Medicine, Democritus University of Thrace, 68100 Alexandroupolis, Greece; pivalsam@med.duth.gr (P.V.); azisimop@med.duth.gr (A.Z.); 4 Department of Clinical Radiation Oncology, Attikon Hospital, School of Medicine, National and Kapodistrian, 12462 Athens, Greece; ikoukourakis@med.uoa.gr; 5 Department of General Surgery, General Hospital of Eastern Achaia-Unit of Aigio, 25100 Aigio, Greece; med5507@ac.upatras.gr; 6 Department of Oncology, University Hospital of Patras, 26504 Patras, Greece; 7 Department of Pathology, School of Medicine, Democritus University of Thrace, 68100 Alexandroupolis, Greece; agiatrom@med.duth.gr

**Keywords:** head–neck cancer, radiotherapy, PET/CT, staging, planning

## Abstract

Background/Objectives: Precise staging and tumor delineation are essential for optimizing treatment and enhancing outcomes of radiotherapy (RT). While computed tomography (CT)-based RT remains standard, fluorine-18-fluorodeoxyglucose positron emission tomography/computed tomography (18F-FDG PET/CT) offers improved detection of primary and nodal disease. This study investigates the role of PET/CT in RT planning of HNSCC. Methods: Fifty-one HNSCC patients underwent radical volumetric modulated arc RT with concurrent cisplatin chemotherapy in a prospective study. Two RT plans per patient were sequentially created by a single oncologist using CT-only and PET/CT data, respectively. Planning target volumes (PTVs) for primary and nodal regions were independently defined, and dose–volume histograms were analyzed and compared. Results: PET/CT significantly affected TNM staging, increasing the T-stage in 11.8% of patients and the N-stage in 33.3%. Distant metastases were found in 9.8% of patients, leading to a redefinition of the overall treatment policy. PET/CT-based planning improved primary tumor PTV coverage (PTV4) in 37.2% (19/51) of cases. The tumor areas excluded from the CT-based planning received an average of 85.6% of the prescribed PTV4 dose (range 18–93%), while in PET/CT planning, they received 95.4% (range 93–99%) (*p* < 0.0001). Nodal PTV areas requiring a booster dose (PTV2) were adjusted in 33.3% (17/51) of patients during PET-CT planning. These nodal areas received an average of 85.6% of the prescribed dose for PTV2 (range 18–93%) during CT planning, compared to 95.4% (range 93–99%) during PET/CT planning. There was no statistically significant difference in the dose received by organs at risk between CT- and PET/CT-RT planning. Conclusions: PET/CT improves target delineation for primary tumors and lymph nodes, also allowing for dose escalation in metabolically highly active lesions in patients with HNSCC. The method also reveals occult distant metastases in a subset of patients, enabling personalized treatment strategies.

## 1. Introduction

Head and neck squamous cell carcinoma (HNSCC) encompasses cancers in various parts of this region, including the oral cavity, pharynx, hypopharynx, larynx, nasal cavity, and salivary glands. It ranks as the seventh most common cancer globally, with approximately 890,000 new cases and 450,000 deaths each year [1]. By 2030, the global incidence of HNSCC is projected to rise by roughly 30% each year, creating a significant worldwide health issue, especially among younger populations. This trend is associated with changes in lifestyle behaviors, such as smoking, alcohol consumption, and human papillomavirus (HPV) infection [2].

Early diagnosis of disease stage and pathological features is essential for guiding treatment decisions and improving survival outcomes in patients with HNSCC. Generally, stages I–II cancer involve a small primary tumor with or without limited nodal spread, depending on the primary site, while stages III–IV may include larger tumors invading nearby structures and extensive regional node involvement. Distant metastases are less common than in lung or esophageal malignancies [3].

According to the National Comprehensive Cancer Network (NCCN), radiotherapy is recommended for 30–40% of patients with early-stage disease (stage I or II). In contrast, locally advanced tumors are usually treated with chemo-radiotherapy (CRT) [4].

Unfortunately, locoregional recurrence is common in patients with locally advanced HNSCC, occurring in 50–70% of cases after CRT. Advances in radiation techniques, such as intensity-modulated radiation therapy (IMRT), have reduced toxicity but do not show a clear benefit in locoregional control [5]. Immunotherapy, on the other hand, has failed to improve the effectiveness of radical CRT, although a significant benefit has been documented when added to post-operative CRT [6,7].

Accurate radiotherapy (RT) planning depends on a detailed understanding of the disease’s local extent and nodal spread to avoid recurrences. Computed tomography-based radiotherapy planning (CT-based RT) continues to be the standard method. 18Fluorine-Labeled Fluorodeoxyglucose Positron Emission Tomography (PET/CT) has proven effective in detecting small cancerous areas that traditional computed tomography (CT) or magnetic resonance imaging (MRI) might overlook, and its use in clinical radiation therapy is recommended [8,9,10]. This enhances the accuracy of target volume delineation for the primary tumor and nodal regions, ultimately contributing to a reduction in locoregional recurrence rates [11]. Furthermore, discovering distant metastases with PET/CT would change the overall treatment plan for patients. PET/CT data analysis of glucose uptake and clearance rates may also have radiobiological value, as it reflects tumor cell proliferation, apoptosis, hypoxia, and angiogenesis. This can aid translational research focused on collecting predictive information and recommending radiation dose adjustments [12,13,14].

This study aims to evaluate how PET/CT imaging influences target volume delineation in radiation therapy planning and the overall management of patients with HNSCC.

## 2. Materials and Methods

A prospective analysis was carried out comparing primary and nodal planning target volumes (PTVs), TNM changes, and PTV dose coverage between CT-based and PET/CT-guided plans. These plans were performed sequentially on the same patients. The study, conducted from April 2023 to September 2025, included 51 patients with histopathologically confirmed HNSCC who were referred to CRT at the Radiotherapy/Oncology Department of the University Hospital of Alexandroupolis. The study was conducted in collaboration with the Nuclear Medicine Department at the University Hospital of Alexandroupolis. The inclusion criteria comprised patients with a good performance status (PS 0–1), histologically confirmed HNSCC, normal renal and hepatic function, and no evidence of distant metastasis on routine CT scans. Patients with severe renal, liver, cardiovascular, or psychiatric diseases, or with a poor performance status (PS ≥ 2), were excluded. The participants were scheduled to receive radical chemo-radiotherapy with cisplatin. The patient characteristics are shown in Table 1. The patients’ ages ranged from 50 to 91, with a mean age of 67.10 years. The 8th edition of the American Joint Committee on Cancer—Tumor Nodes Metastasis (AJCC TNM) system was used for HNSCC cancer staging [15].

The Ethics and Research Committee of the University Hospital of Alexandroupolis approved the study (No. ES1 12-01-2023). All patients provided written informed consent, authorizing the anonymous use of their clinical and laboratory data for research and publication.

### 2.1. Staff Training

Properly trained staff are vital for maintaining quality assurance and procedure safety. PET/CT-based RT necessitates collaboration between technical and medical staff in nuclear imaging and radiation oncology departments. All personnel involved in the project received training on patient setup and imaging protocols to prevent errors and conflicts. Standard PET/CT patient preparation was carried out. Additionally, patients were thoroughly informed and prepared for the procedures to ensure an effective PET/CT-guided RT simulation.

### 2.2. PET/CT Simulation

The patients were positioned supine on a carbon fiber couch, with arms at their sides and immobilized using a 5-point thermoplastic mask. Radio-opaque fiducial markers were placed according to the disease’s anatomical location, guided by three perpendicular laser beams installed in the treatment room.

PET/CT scans were performed using the ${Discovery}^{TM}$ MI PET/CT system (General Electric Healthcare, Boston, MA, USA, 2022), which includes a 4-ring PET system with LightBurst digital detectors and a 64-slice CT unit. The CT uses a tube voltage of 120 kV, automatic Smart tube current modulation from 100 to 300 mA, and a slice thickness of 3.75 mm for attenuation correction and anatomical localization. The imaging settings included a pitch factor of 0.984:1, a speed of 39.37 mm/s, a rotation time of 0.5 s, a maximum Field of View (FOV) of 70 cm, a detector coverage of 40 mm, and a coverage speed of 78.75 mm/s.

A 90 s bed position technique was used to acquire the PET images, covering from the mid-thigh to the top of the skull. Using a 512 × 512 matrix and a Gaussian post-filter for smoothing and sharpening, iterative reconstruction algorithms of OSEM (Ordered Subsets Expectation Maximization) with time-of-flight (TOF) and point spread function (PSF) correction were employed to reconstruct the image.

The tracer 18F-FDG was administered intravenously at a dose ranging from 2.2 to 2.5 MBq/kg (0.059 to 0.067 mCi/kg) by a qualified nuclear physician. The mean ± SD dose administered was 4.85 ± 0.99 mCi. Each patient remained in the waiting room for 60 min to ensure proper tracer distribution and uptake in the target tissues. The radiotracer uptake area was properly shielded to reduce radiation exposure for healthcare professionals and other patients.

Before imaging, all patients fasted for at least 4–6 h to keep serum glucose levels low. Serum glucose was checked before the radiotracer injection to ensure it was within the acceptable range (70–150 mg/dL). The average ± standard deviation (SD) levels were 115.10 ± 14.58 mg/dL. The patients were advised to stay well-hydrated with water and avoid strenuous physical activity before the scan. All procedures followed institutional ethical guidelines, and informed consent was obtained from all participants.

### 2.3. PET-CT Interpretation

An experienced nuclear physician reviewed all PET images. Qualitative assessment was complemented by a quantitative analysis, with the maximum standardized uptake value (SUVmax) measured for each tumor lesion by drawing a rectangular region of interest (ROI) that included the entire tumor volume. Typically, malignancies are defined as areas of abnormal FDG uptake with standardized uptake values (SUVs) of 2.5 or higher. Some malignant tumors have relatively low metabolic activity and may show SUVs of ≤2.5 on FDG PET/CT. Therefore, an SUV threshold of ≥2.5 should be viewed as a probabilistic or statistical indicator of malignancy rather than an absolute diagnostic criterion, and it should be interpreted alongside anatomical imaging findings, tumor histology, and the overall clinical context [16]. Less intense 18F-FDG uptake was also considered suspicious for a tumor, especially when it matched a CT-evident abnormality. The mean SUVmax of lesions was 12.08 ± 7.25 on 18F-FDG PET/CT images. PET/CT-based segmentation was performed manually on fused, attenuation-corrected PET/CT images using an SUV threshold of ≥2.5 and visual assessment.

### 2.4. Radiotherapy Schedule Details

All patients were treated with the volumetric modulated arc therapy VMAT technique, using a 6-MV Elekta Infinity Linear Accelerator (Elekta, Stockholm, Sweden) equipped with an Agility head (Elekta). The treatment plans were created using Monaco TPS version 6.1.4.0 (Elekta CMS, Maryland Heights, MO, USA). For VMAT we used 2 Arcs of 360 degrees (with 150 Control Points per Arc, 12 sectors of 30 degrees and statistical uncertainty 1% per calculation). Patient-specific quality assurance (PSQA) was conducted before clinical delivery using the Delta4+ diode array phantom (Scandidos, Uppsala, Sweden). Measured and calculated dose distributions were compared using gamma analysis with criteria of 3%/3.0 mm. A gamma passing rate of ≥95% was considered acceptable.

The dose was prescribed to the International Commission on Radiation Units and Measurements ICRU reference point for head and neck cancer. The plans were optimized to maximize the dose of the PTV while minimizing the dose of the surrounding normal tissues. Image-guided RT using cone-beam computed tomography (CBCT), before each radiation treatment, was performed by the Elekta platform Synergy kV CBCT (XVI) to assess and adjust the patients’ positions.

Our department employs a simultaneous integrated boost technique for radical RT in HNSCC patients to accelerate treatment. Treatment is concluded within 28 consecutive fractions. Four distinct PTVs are identified and treated with different RT fractionation as follows. PTV4 includes the primary tumor area recognized on PET/CT, which receives 2.30–2.35 Gy per day. PTV3 includes the high-risk area around the primary tumor that receives 2.20 Gy per day. A margin of 0.5cm with manual correction around the CTV3 and CTV4 was considered for PTV3 and 4. PTV2 covers nodal regions identified as involved or at high risk in PET-CT, receiving a dose of 2.15 Gy per day. Within this PTV2 area, large nodes that are clearly identified received a daily dose of 2.2–2.35 Gy, with a small margin of 0.3 cm around node CTV. PTV1 covers neck areas at low risk of recurrence that receive 1.8 Gy per day.

### 2.5. Chemotherapy Details

All patients underwent concurrent chemotherapy with cisplatin (60 mg/m^2^) every two weeks, starting one week before the onset of radiotherapy. A total of four cycles have been administered, delivering a total dose of 240 mg of cisplatin during RT. To prevent cisplatin-induced anemia, intravenous administration of iron was considered for hemoglobin levels below 12 g/dL. The patients who developed grade II-III neutropenia were treated with pegylated recombinant human granulocyte colony-stimulating factor (rhG-CSF), and chemotherapy was delayed by one week without interrupting RT. None of the patients developed grade IV or febrile neutropenia.

### 2.6. Radiotherapy Treatment Planning

Each PTV was planned to deliver 95% of the total dose to 98% of the minimum volume, ensuring sufficient coverage, and to deliver 100% of the total dose to 50% of the minimum volume to control the mean dose to the target. The quadratic overdose cost function was used to control the maximum dose of PTVs and was set at 100% of the maximum dose, with a root mean square (RMS) of 50 cGy.

Radiotherapy treatment planning was performed the day after the PET-CT simulation. An experienced radiation oncologist specializing in head and neck cancer developed two separate treatment plans for each patient. The same physician carried out the planning for all the patients included in the study. PET/CT-based segmentation was performed manually on fused, attenuation-corrected PET/CT images using an SUV threshold of ≥2.5 and visual assessment. Manual Contouring was carried out by an experienced radiation oncologist in collaboration with a nuclear medicine physician, with interpretation guided by CT anatomy and the patient’s clinical history.

The spatial resolution of the CT images was 1 mm, while the intrinsic spatial resolution of the PET images was approximately 4 mm. In this study, the radiotherapy planning process was carried out in two sequential steps. In the initial step, treatment planning relied solely on the high-resolution CT dataset, which was used for initial target delineation and creating the primary radiotherapy plan. In the second step, co-registered PET and CT images were integrated, combining CT’s high anatomical resolution with the metabolic information from PET after attenuation correction. This fused PET/CT dataset was then used to refine target delineation and develop the second radiotherapy plan. Dose–volume histograms were calculated separately for the PTV and organs at risk (OARs), and the plans were approved for dosimetric analysis.

### 2.7. Target Volume Delineation

Planning CT and PET images were transferred as DICOM files to the Treatment Planning System (TPS) for structure delineation and treatment plan creation [17]. In the first plan, gross tumor, clinical target, and planning target volumes were defined solely based on the anatomical data provided by CT (GTV_CT, CTV_CT, PTV_CT), and in the second, on images fused from PET/CT data (GTV_PET-CT, CTV_PET-CT, PTV_PET-CT).

Target volume and organs at risk were contoured following ESTRO ACROP guidelines [18]. After delineating the primary tumor and involved node GTV, a 0.5 cm margin was added to create the CTV. An extra margin of 0.2–0.5 cm, adjusted manually based on the proximity to critical anatomical structures, was used to define the PTVs.

The organs at risk (OARs) follow the guidelines of the Radiation Therapy Oncology Group (RTOG) 0225/0615 protocols for HNSCC IMRT [19]. Dose constraints for each organ were applied using the QUANTEC criteria adapted for head and neck treatment [3]: Brainstem Dmax < 54 Gy; PRV-Spinal cord Dmax < 50 Gy; Optic chiasm Dmax < 55 Gy; Mandible (1 cc) < 70 Gy; Optic nerve Dmax < 55 Gy; Parotid gland Dmean < 26 Gy; Cochlea Dmean < 45 Gy; Eye Dmax < 50 Gy; and Lip Dmean < 30 Gy. The maximum dose to the brachial plexus and the mean dose to dysphagia/aspiration-related structures (DARSs) were recorded, but no specific dose constraints were applied [20].

### 2.8. Documentation of Response

Response to CRT was documented with CT or MRI scans performed 2 months after the end of RT. We used the RECIST 1.1 criteria to record response [21]. Complete Response (CR) referred to the elimination of all detectable disease. Partial response (PR) was defined as a decrease in the sum of the longest diameters (of all irradiated lesions) by more than 30%. Progressive disease (PgD) was documented as an increase of >20% in the longest dimension. All other cases were recorded as stable disease (SD).

### 2.9. Statistical Analysis

Data collected were analyzed using standard statistical methods, and the Wilcoxon matched-pairs signed-rank test was used to assess significance. Analysis was performed to compare the means of groups using SPSS v29.0.0 [22] and GraphPad Prism 8.1. [23]. Overall disease-specific survival (OS) and locoregional progression-free survival (LPFS) analysis were performed using the GraphPad Prism version 8.1 statistical package and the Kaplan–Meier method. LPFS and OS were defined as the time from the start of RT to the first documentation of locoregional progressive disease or death from cancer. A *p*-value of <0.05 was considered statistically significant.

## 3. Results

### 3.1. Changes in Patient Staging

The PET/CT scan showed a significant change in TNM staging. Specifically, PET/CT resulted in T-stage upstaging in 11.8% (6/51) of the participants and downstaging in 1.9% (1/51). N-upstaging occurred in 33.33% (17/51), while downstaging was seen in 3.92% (2/51). Additionally, PET-CT identified distant metastasis in 9.80% (5/51) of the cases. Table 2 offers an overview of the TNM redistribution.

### 3.2. Comparison Between RT-Plans—Primary Tumor and Related Area

**Figure 1:**
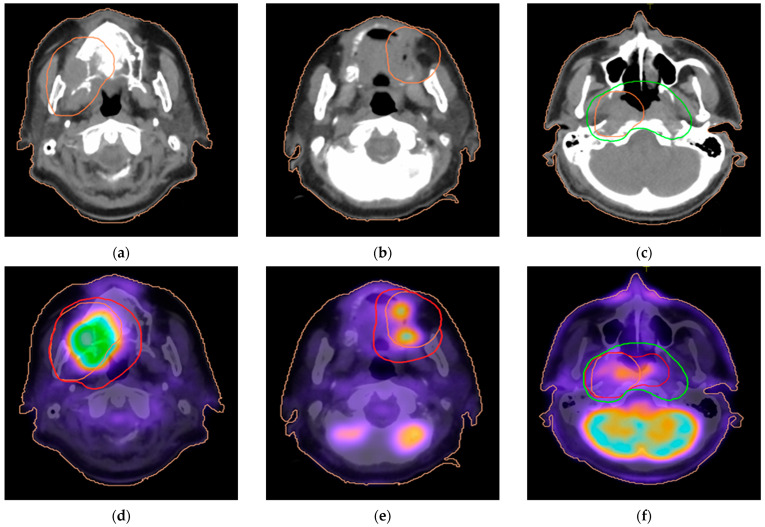
Examples of improvement in primary tumor coverage after PET/CT-based radiotherapy planning. Inadequate coverage of the primary tumor area (orange line; PTV4) based on CT treatment planning (**a**–**c**). PTV4 (red line) was extended to encompass the tumor visible in PET-CT (**d**–**f**). The green line shows the PTV3 margins.

**Figure 2:**
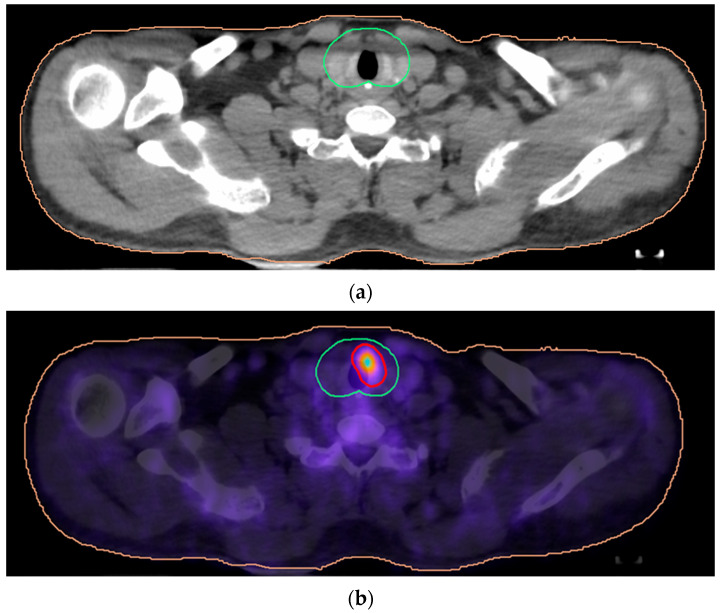
Examples of improvement in primary tumor coverage after PET/CT-based radiotherapy planning by adding a boost. Contouring a primary tumor area (green line; PTV1) based on CT treatment planning (**a**). A PTV4 area was added to extend primary tumor coverage by including a boost based on PET/CT-detected paratracheal involvement (**b**).

Integrating PET/CT imaging into radiotherapy planning resulted in an increase in the planning target volume (PTV) margins in 19 of 51 (37.2%) cases to ensure adequate coverage of the primary tumor. In these cases, the coverage of tumor areas shifted from PTV3 to PTV4, thereby increasing the radiation dose to those regions. It is worth noting that in some cases (19/51 37.2%), the PTV expansion was necessary due to metabolic evidence of tumor extension into adjacent anatomical structures, including the ethmoid sinus (3/51), trachea/and paratracheal space (4/51), nasopharynx (6/51), hard palate (2/51), and subglottic space (4/51). In one patient, the tumor was not visible in CT due to dental implant artifacts but was clearly visible in PET/CT images. In one additional patient, the PTV4 volume decreased due to dose-sparing constraints for the optic nerves and the ethmoid region after PET/CT documented a lack of invasion. Table 3 summarizes observed modifications of primary target volumes. Figure 1 and Figure 2 display an example of insufficient coverage of the primary tumor area, which was corrected after PET/CT planning.

PTV4 coverage of the primary tumor area, defined as the percentage of patients receiving at least 95% of the prescribed dose (V95%), was significantly increased in PET/CT planning. The mean PTV4 V95% increased to 95.97 ± 0.99%, compared to 90.18 ± 15.15% for CT-based plans (*p* = 0.007).

We further compared the mean dose delivered to the PTV4 area included in the corrected PET/CT planning, which was not part of the CT planning, for the 19 patients with corrections based on PET/CT data. By subtracting the CT-PTV4 from the PET/CT-PTV4, the dose received by this specific tumor area was analyzed. The 95% of the volume of this subtracted tumor area received a median of 95.4% (range 93–99%) of the dose prescribed to PTV4 in the PET/CT-planning vs. 85.6% (range 18–93%) in the CT-planning. This was statistically significant (*p* < 0.0001).

### 3.3. Comparison Between RT-Plans—Involved Nodes and Neck Area

Regarding nodal disease, PET/CT findings prompted verification of lymph node involvement, allowing for more accurate detection of nodal metastases and providing crucial information for treatment planning.

PET/CT showed unexpected nodal involvement in neck areas not identified on the CT scan in six patients (6/51, 11.8%). Specifically, 1/6 of the patients demonstrated lymphadenopathy in the supraclavicular region, 3/6 had ipsilateral cervical lymph nodes involved, and 2/6 showed paraesophageal lymph node involvement. For these patients, the involved neck areas were included in PTV2 instead of PTV1, increasing the radiation dose to high-risk regions for recurrence.

**Figure 3:**
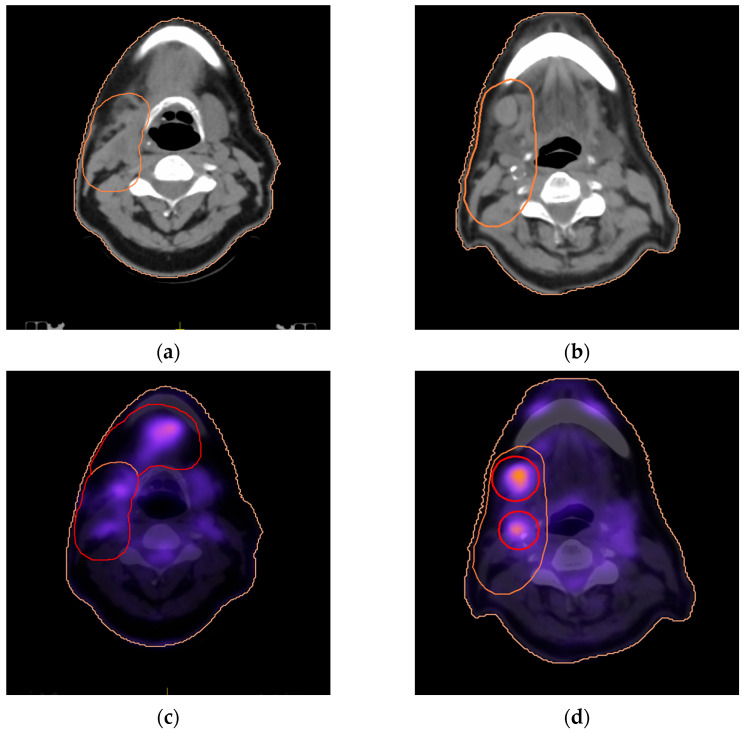
Examples of PET/CT-guided radiotherapy planning showing extended PTV2 area to cover submandibular extension undetected in the CT scan (orange line extended to red line) (**a**,**c**). Delineation of metabolically active nodes to receive a booster dose within the PTV2 area (**b**,**d**).

Furthermore, PET/CT imaging allowed more precise delineation of PTV2 in 17 out of 51 (33.3%) patients by better identifying the extent of metabolically active lymphadenopathy within the high-risk neck regions. It also helped contour the PTV for large nodes by delivering an extra targeted radiation dose solely to metabolically active nodal regions, as described in the methods, in 4/51 cases (7.84%). Table 3 summarizes observed modifications of nodal target volumes. Figure 3 shows a typical image where an increased RT dose was planned for a metabolically active large node area.

We further compared the mean dose delivered to the PTV2 area (nodal areas that received the prescribed highest dose) included in the corrected PEΤ/CT planning, which was not part of the CT planning, for the 17 patients with corrections based on PET/CT data. By subtracting CT-PTV2 from PET/CT-PTV2, the dose received by this specific nodal area was analyzed. The 95% of the volume of this subtracted tumor area received a median of 96.6% (range 92–100%) of the dose prescribed to PTV2 in the PET/CT-planning vs. 84.4% (range 13–89%) in the CT-planning. This was statistically significant (*p* < 0.0001).

### 3.4. Change in Overall Therapy Plan

In five patients (10%), PET/CT identified metastatic lesions in the lungs (4/51 patients) and bones (1/51 patients) that were not seen on conventional CT scan. For these patients, the overall treatment plan was adjusted to include systemic chemotherapy for metastatic disease after chemo-RT and the delivery of SBRT to metastatic lesions. The chemotherapy regimen used was a combination of cisplatin 60 mg/m^2^ and a 48 h infusional administration of 5-fluorouracil (1000 mg/m^2^/day) via a port-a-cath using a portable pump. Chemotherapy was delivered every two weeks.

### 3.5. Changes in the Primary Tumor and Nodal Volumes

**Figure 4:**
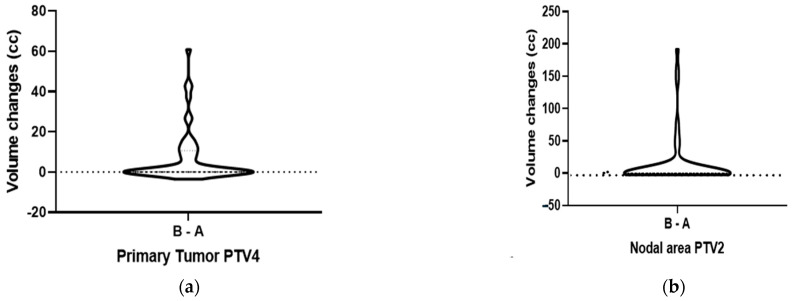
Modification of Planning Treated Volume (PTV) in cc of the primary tumor PTV4 (**a**) and lymph node PTV2 (**b**) in CT-based and PET/CT-based planning.

We further examined the volume changes (in cc) of the PTVs for the primary tumor and lymph node regions by comparing CT- and PET/CT-based RT planning. Detailed information on the primary target PTV and lymph node PTV, based on CT scan and PET/CT scan, including volume differences and percentage volume differences (in %) for each patient, is shown in Appendix A. Overall, the mean (±standard deviation, SD) PTV4 increased from 111 ± 108 cc to 118 ± 110 cc (*p* < 0.001); Figure 4a. The mean (±SD) PTV2 increased from 272 ± 199 cc to 286 ± 199 cc (*p* < 0.018); Figure 4b. For both the primary tumor and lymph node PTVs, the data were not normally distributed according to the Shapiro–Wilk test (primary: W = 0.842, *p* < 0.0001; lymph nodes: W = 0.948, *p* = 0.026). Therefore, the Wilcoxon matched-pairs signed-rank test was used to assess significance.

### 3.6. Dose at Organs at Risk

Table 4 shows the mean dose to organs at risk (OARs) recorded at the original CT-based RT-planning vs. the corrected planning on PET/CT images. There was no statistically significant difference in the dose received between CT- and PET/CT-RT planning.

### 3.7. Response to Therapy and Survival Analysis

Two patients died before completing treatment for reasons unrelated to the tumor or therapy, and one refused further radiotherapy before finishing treatment. Table 5 presents the tumors’ responses to RT by primary location.

**Figure 5:**
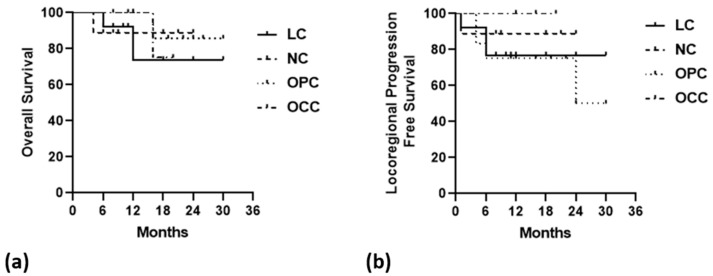
Kaplan–Meier disease-specific overall survival (**a**) and locoregional progression-free survival (**b**) stratified by the four major primary tumor locations (LC = laryngeal cancer, NC = nasopharyngeal cancer, OPC = oropharyngeal cancer, OCC = oral cavity cancer).

Kaplan–Meier disease-specific OS and LPFS, stratified by the four main primary tumor location subgroups (laryngeal, nasopharyngeal, oropharyngeal, and oral cavity cancers), are shown in Figure 5.

## 4. Discussion

Locoregional recurrence remains the primary cause of failure after radiotherapy in patients with HNSCC. The use of innovative imaging methods like 18F-FDG PET/CT in advanced radiation techniques such as IMRT/VMAT has ushered in a new era of highly precise radiation therapy. These advanced techniques enable more accurate delineation of target volumes, including the primary tumor and involved nodal regions, and can also reduce radiation exposure to surrounding healthy tissues in some cases.

The use of 18FDG-PET/CT is considered part of the initial diagnostic process for HNSCC according to international guidelines [4,24,25], and it has recently been integrated into adaptive radiotherapy strategies. PET/CT assists in detecting primary tumor extensions in HNSCC and undifferentiated nasopharyngeal tumors, with reported overall sensitivity ranging from 93% to 100%, specificity from 90% to 100%, and accuracy between 94% and 98% [25,26]. Our study showed a significant change in TNM staging. PET/CT led to T- and N-upstaging in 11.8% and 33.3% of patients, respectively. Additionally, distant metastases that were previously undetected were found in 9.8% of cases. Our observations on TNM stage modifications align with Matsuura et al., who reported that the N-stage was most frequently changed (16%), followed by the M-stage (12%) [25]. Initial research has also validated alterations in TNM staging following PET/CT imaging. In particular, preliminary investigations found PET/CT-wise amendments in TNM categories in 22% [27] and 57% [28] of patients with HNSCC.

Several studies reported that the overall treatment decision and RT fields may be modified in approximately 30% of cases with the incorporation of PET imaging [29,30,31,32,33]. Interobserver variability in volume delineation is reduced, and PET/CT helps identify regions within the volume that might require an additional radiation dose [14]. In our prospective study, a similar percentage of patients (35.3%) had their initial treatment plan modified. These changes included coverage of the primary tumor, identification of high-risk neck regions, and administration of a booster focal dose to large nodes with high metabolic activity. Functional imaging in radiotherapy enables dose-painting-IMRT, allowing the delivery of spatially varying dose distributions tailored to biological tumor heterogeneity. Specifically, the identification of hypoxic lesions via PET-hypermetabolism is crucial since glycolytic cells appear more resistant to radiation in experimental studies [34]. Thus, 18F-FDG PET guides more precise treatment planning, in terms of targeted dose escalation [35]. Regarding nodal disease, PET/CT findings also led to expanding the high-risk area to include involved nodal regions (e.g., paraoesophageal and supraclavicular nodes) that were not visible on CT alone.

The literature reports that PET/CT shows high sensitivity (98%) and specificity (95%) in detecting distant metastatic disease [36,37]. Consistent with these reports, the current study discovered lung metastases in four patients and bone metastases in one patient through PET/CT imaging. Another common site for metastases in HNSCC is the liver, but this was not observed in our study [38]. Among the five patients with metastases, three cases had advanced T-stage tumors (T3–T4), and four patients had nodal involvement. These findings indicate that advanced TN stage is linked to a higher risk of distant metastases.

An important advantage of our study is that it prospectively assesses the impact of PET-CT modifications on radiotherapy treatment planning by a single experienced radiation oncologist who sequentially conducted CT-based and PET/CT-based RT planning. The limitations of our study include the heterogeneity in the sample, which consisted of HNSCCs from various primary sites, while focusing on a single tumor type would enable a more comprehensive analysis. A larger patient cohort would have improved the ability to specify the benefits of PET-CT RT planning in different HNSCC categories. Glucose PET-CT is a well-established method, and the use of tracers that target hypoxia or cancer cell proliferation could offer new insights. However, we believe that demonstrating the value of an existing method in a therapeutic area remains valuable for clinicians, as it promotes incremental progress in current clinical practice.

## 5. Conclusions

18F-FDG PET-CT is an essential imaging tool for radiotherapy planning and overall treatment management in HNSCC patients. It can clearly identify primary tumor extension, involved lymph nodes, and distant metastases, thereby enhancing diagnostic accuracy and individualizing patient treatment. Additional research is needed to clarify the advantages of PET/CT in radiotherapy outcomes for HNSCC. Dose escalation techniques targeting areas of high metabolic activity remain an ongoing challenge for clinical trials.

## Figures and Tables

**Table 1:** Patient, disease characteristics, and CT parameters.

**Feature**	
Patients (number)	51
**Gender**	
Male	37
Female	14
**Patient characteristics**	
Age (years) (median ± SD)	67.10 ± 11.50
Height (cm) (median ± SD)	167.31 ± 9.39
Weight (kg) (median ± SD)	72.54 ± 16.30
**Tumor location**	
Laryngeal cancer	13
Oropharyngeal cancer	12
Nasopharyngeal cancer	9
Oral cancer	7
Hypopharyngeal cancer	5
Paranasal sinus cancer	4
Salivary gland cancer	1
**Histology**	
Squamous cell carcinoma	51
**T-stage (CT-based)**	
T0 (*)	6
T1,2	16
T3	17
T4	12
**N-stage (CT-based)**	
N0	16
N1	14
N2	15
N3	6
**M-stage (CT-based)**	
M0	51
M1	0

Abbreviations: SD: standard deviation; T: tumor; CT: computed tomography; N: nodule; M: metastasis. (*) Three patients had neck metastatic disease from previous squamous cell skin cancer of the face and scalp, two patients had neck metastasis from nasal and laryngeal cancer treated with surgery in the past, and one patient had neck disease of unknown primary.

**Table 2:** TNM-staging of the disease before and after PET/CT.

T-stage-wise distribution of patients				
T-Stage	CT scan	PET-CT scan	Change (percentage change)	
T0	6	6		
T1	1	0	Upstaged	6/51 (11.76%)
T2	15	13	Downstaged	1/51 (1.96%)
T3	17	18	No change	44/51 (86.27%)
T4	12	14		
N-stage-wise distribution of patients				
N-Stage	CT scan	PET-CT scan	Change (percentage change)	
N0	16	6	Upstaged	17/51 (33.33%)
N1	14	16	Downstaged	2/51 (3.92%)
N2	15	19	No change	32/51 (62.75%)
N3	6	10		
M-stage-wise distribution of patients				
M-Stage	CT scan	PET-CT scan	Change (percentage change)	
M0	51	46	Upstaged	5/51 (9.80%)
M1	0	5	Downstaged	0/51 (0%)
			No change	46/51 (90.20%)

Abbreviations: Τ: tumor; Ν: node; Μ: metastasis stage; CT: computed tomography; PET-CT: 18Fluorine-Labeled Fluorodeoxyglucose Positron Emission Tomography.

**Table 3:** Overview of changes made to the RT planning.

VOLUME CHANGES IN RT PLAN	
Category	No. of Patients (%)
PTV4 margin expansion to ensure coverage of the primary tumor	19/51 (37.2)
PTV2 margin expansion to include high-risk neck area	17/51 (33.3)
Inclusion of additional neck regions in PTV2 (undetectable nodes away from the bulky nodal involvement)	6/51 (11.8)
Increase in radiation dose to large metabolically active nodes within PTV2	4/51 7.84
Identification of metastatic disease	5/51 (10)

Abbreviations: PTV4: Planning Tumor Volume of the primary tumor area; PTV2: Planning Tumor Volume of the nodal area.

**Table 4:** Mean dose to organs at risk (OARs) recorded at the original CT-based RT plan vs. the corrected plan on PET/CT images. For the spinal cord and eyes, the ‘mean value’ of the RT dose refers to the average of the maximum doses received by patients. For all other organs, the ‘mean value’ of RT dose represents the average of the mean doses received by patients.

	Mean Value (cGy)		
Organ at Risk	CT Planning	PET/CT Planning	*p*-Value
Spinal cord	3187	3457	0.23
Mouth	1424	1475	0.75
Larynx	1911	2004	0.11
Left parotid	2213	2204	0.34
Right parotid	2212	2224	0.81
Left submandibular	2640	2652	0.51
Right submandibular	2602	2662	0.10
Left eye	1593	1589	0.93
Right eye	1441	1444	0.95

Abbreviations: cGy: centigray; CT: computed tomography; PET/CT: 18Fluorine-Labeled Fluorodeoxyglucose Positron Emission Tomography.

**Table 5:** Response of the tumor to therapy according to the RECIST 1.1 criteria. Three patients were not assessable (na).

Primary Tumor Location	No Cases	CR	PR	SD	PD	Na
Laryngeal cancer	13	10 (76.9)	2 (15.4)	0 (0)	1 (7.7)	0
Oropharyngeal cancer	12	9 (75.0)	3 (25.0)	0 (0)	0 (0)	0
Nasopharyngeal cancer	9	7 (77.8)	2 (22.2)	0	0 (0)	0
Oral cancer	7	6 (85.7)	0 (0)	0 (0)	0 (0)	1
Hypopharyngeal cancer	5	2 (40.0)	1 (20.0)	1 (20.0)	0 (0)	1
Paranasal sinus cancer	4	0 (0)	2 (50.0)	1 (25.0)	0 (0)	1
Salivary gland cancer	1	0 (0)	1 (100)	0 (0)	0 (0)	0

Abbreviations: CR: Complete Response; PR: partial response; SD: stable disease; PD: progressive disease; Na: not assessable.

## Data Availability

Data is unavailable due to privacy or ethical restrictions.

## Abbreviations

The following abbreviations are used in this manuscript:

HNSCC	head and neck squamous cell carcinoma
NCCN	National Comprehensive Cancer Network
CRT	chemo-radiotherapy
IMRT	intensity-modulated radiation therapy
RT	radiotherapy
CT-based RT	computed tomography-based radiotherapy planning
PET/CT	18Fluorine-Labeled Fluorodeoxyglucose Positron Emission Tomography
CT	computed tomography
MRI	magnetic resonance imaging
PTV	planning target volume
TNM	Tumor Nodules and Metastasis
PS	performance status
AJCC TNM	American Joint Committee on Cancer—Tumor Nodes Metastasis
FOV	Field of View
OSEM	Ordered Subsets Expectation Maximization
TOF	time-of-flight
PSF	point spread function
SD	standard deviation
SUVmax	Maximal Standardized Uptake Value
ROI	region of interest
SUVs	standardized uptake values
^18^F-FDG	Fluorodeoxyglucose Labeled With Fluorine-18
VMAT	volumetric modulated arc therapy
ICRU	International Commission on Radiation Units and Measurements
CBCT	cone-beam computed tomography
rhG-CSF	recombinant human granulocyte colony-stimulating factor
PSQA	patient-specific quality assurance
OARs	organs at risk
TPS	Treatment Planning System
GTV	Gross Tumor Volume
CTV	Clinical Target Volume
RMS	root mean square
QUANTEC	Quantitative Analyses of Normal Tissue Effects in the Clinic
Dmax	dose maximum
Dmean	dose mean value
DARS	dysphagia/aspiration-related structures
PR	partial response
PgD	progressive disease
SD	stable disease
CR	Complete Response
OS	overall disease-specific survival
LPFS	locoregional progression-free survival

## Appendix A

**Table A1:** Comparison of primary and lymph nodes PTV (volume in cc) for CT versus PET/CT RT planning.

	Primary Tumor				Nodal Area			
No. Patient	Primary PTV on CT Scan (cc)	Primary PTV on PET/CT Scan (cc)	Volume Difference (cc)	Percentage Volume Difference (%)	Nodal PTV on CT Scan (cc)	Nodal PTV on PET/CT Scan (cc)	Nodal Volume Difference (cc)	Nodal Percentage Volume Difference (%)
1	62.10	62.10	0.00	0.00%	283.48	283.48	0.00	0.00%
2	40.74	48.95	8.21	20.15%	0.00	0.00	0.00	0.00%
3	0.00	0.00	0.00	0.00%	900.69	900.69	0.00	0.00%
4	89.19	89.19	0.00	0.00%	610.68	610.68	0.00	0.00%
5	38.71	41.26	2.55	6.59%	287.59	287.59	0.00	0.00%
6	390.20	390.20	0.00	0.00%	0.00	0.00	0.00	0.00%
7	327.28	327.28	0.00	0.00%	617.99	617.99	0.00	0.00%
8	75.20	75.20	0.00	0.00%	0.00	0.00	0.00	0.00%
9	0.00	0.00	0.00	0.00%	305.94	448.41	142.48	46.57%
10	0.00	0.00	0.00	0.00%	333.25	333.25	0.00	0.00%
11	45.74	54.29	8.55	18.68%	260.21	260.21	0.00	0.00%
12	0.00	0.00	0.00	0.00%	114.49	153.84	39.35	34.37%
13	54.14	115.01	60.87	112.44%	0.00	191.88	191.88	0.00%
14	72.35	72.35	0.00	0.00%	123.50	123.50	0.00	0.00%
15	137.75	137.75	0.00	0.00%	407.38	407.38	0.00	0.00%
16	52.58	63.40	10.82	20.58%	152.03	152.03	0.00	0.00%
17	151.37	165.75	14.39	9.50%	294.69	303.37	8.68	2.95%
18	147.52	147.52	0.00	0.00%	167.89	167.89	0.00	0.00%
19	0.00	0.00	0.00	0.00%	239.86	239.86	0.00	0.00%
20	0.00	0.00	0.00	0.00%	267.53	267.53	0.00	0.00%
21	42.66	42.66	0.00	0.00%	79.49	141.05	61.56	0.00%
22	27.37	27.37	0.00	0.00%	0.00	79.13	79.13	0.00%
23	63.22	63.22	0.00	0.00%	421.14	421.14	0.00	0.00%
24	111.90	149.07	37.17	33.22%	251.81	251.81	0.00	0.00%
25	341.60	341.60	0.00	0.00%	0.00	0.00	0.00	0.00%
26	112.96	156.71	43.75	38.73%	313.10	313.10	0.00	0.00%
27	112.82	112.82	0.00	0.00%	425.39	425.39	0.00	0.00%
28	25.57	25.57	0.00	0.00%	125.31	125.31	0.00	0.00%
29	55.21	82.88	27.67	50.11%	188.14	188.14	0.00	0.00%
30	34.93	34.93	0.00	0.00%	408.71	408.71	0.00	0.00%
31	131.48	131.48	0.00	0.00%	295.79	295.79	0.00	0.00%
32	76.20	81.55	5.35	7.02%	213.97	213.97	0.00	0.00%
33	0.00	0.00	0.00	0.00%	78.00	126.05	48.05	61.60%
34	133.91	133.91	0.00	0.00%	198.37	198.37	0.00	0.00%
35	205.84	205.84	0.00	0.00%	139.31	139.31	0.00	0.00%
36	173.30	173.30	0.00	0.00%	370.11	370.11	0.00	0.00%
37	130.21	130.21	0.00	0.00%	385.01	385.01	0.00	0.00%
38	201.51	201.51	0.00	0.00%	484.22	484.22	0.00	0.00%
39	147.96	147.96	0.00	0.00%	408.22	408.22	0.00	0.00%
40	129.89	155.54	25.66	19.75%	365.84	365.84	0.00	0.00%
41	94.03	94.03	0.00	0.00%	235.28	235.28	0.00	0.00%
42	47.42	47.42	0.00	0.00%	0.00	0.00	0.00	0.00%
43	44.28	50.92	6.64	14.98%	333.71	333.71	0.00	0.00%
44	111.54	128.65	17.11	15.34%	342.79	342.79	0.00	0.00%
45	412.55	428.00	15.45	3.74%	384.17	381.62	−2.54	-0.66%
46	149.03	161.46	12.44	8.34%	584.85	746.28	161.44	27.60%
47	38.72	49.40	10.67	27.56%	656.02	656.02	0.00	0.00%
48	76.44	72.88	-3.56	−4.65%	0.00	0.00	0.00	0.00%
49	463.17	475.74	12.57	2.71%	361.24	361.24	0.00	0.00%
50	99.29	140.89	41.59	41.89%	0.00	0.00	0.00	0.00%
51	217.08	219.92	2.84	1.31%	463.08	463.08	0.00	0.00%
**Mean**	111.7	118.8	7.07		272.16	286.48	14.31	
**Standard deviation**	108.9	110.2	13.51		199.85	199.59	41.65	
